# Comparative Evaluation between Visual and Automated Dipstick Urinalyses in Dogs

**DOI:** 10.3390/vetsci10040284

**Published:** 2023-04-10

**Authors:** Erasmia D. Smyroglou, Labrini V. Athanasiou, Rania D. Baka, Zoe S. Polizopoulou

**Affiliations:** 1 Diagnostic Laboratory, Companion Animal Clinic, School of Veterinary Medicine, Faculty of Health Sciences, Aristotle University of Thessaloniki, 54627 Thessaloniki, Greece; erasmiavet@gmail.com (E.D.S.); raniadbaka@gmail.com (R.D.B.); 2 Clinic of Medicine, Faculty of Veterinary Medicine, School of Health Sciences, University of Thessaly, Trikalon 224, 43100 Karditsa, Greece; lathan@vet.uth.gr

**Keywords:** dipstick, dog, urine, automated, visual

## Abstract

**Simple Summary:**

The purpose of the current study was to compare visual and automated evaluations of dipstick variables in canine urine samples. Urine dipstick chemical reaction is based on the color change of a reagent when a specific substance is detected. The automated analysis of urine dipsticks has improved the accuracy and precision of the results.

**Abstract:**

Urine test strips are commercially available and can be assessed with semi-automated analyzers or by visual assessment. This study aimed to compare the visual and automated evaluations of dipstick variables in canine urine samples. One hundred and nineteen urine samples were evaluated. Automated analysis was performed on a veterinary urine analyzer URIT-50Vet (URIT Medical Electronic) with UC VET13 Plus strips. Multistix 10 SG dipsticks (Siemens Healthcare GmbH, Erlangen, Germany) were used for visual evaluation, along with a refractometer (Clinical Refractometer Atago T2-Ne, Atago Co., Tokyo, Japan) for urine specific gravity measurements. A linear relationship was observed between the pH measurements (*p* = 0.2) of the two methods; the Passing–Bablok procedure was valid since neither proportional nor systematic significant errors were observed. Comparing the two methods, the correlation for urine specific gravity was poor (*p* = 0.01, CI 0.667–1.000). Moderate agreement was demonstrated for proteins (κ = 0.431), bilirubin (κ = 0.434) and glucose (κ = 0.450). Agreement was substantial for blood (κ = 0.620) and poor for leukocytes (κ = 0.100). Poor agreement was observed for ketones (κ = −0.006). Apart from the pH analysis, visual and automated dipstick urinalyses should not be used interchangeably. Multiple urine samples obtained from the same dog during the day should be evaluated using the same method to overcome erroneous results.

## 1. Introduction

Urinalysis is a minimally invasive and low-cost diagnostic tool that can provide useful information to the veterinarian. It contributes to the diagnosis of urinary system diseases and liver diseases (e.g., liver failure), diabetes mellitus/insipidus and hemolysis. Urinalysis results may be influenced by the primary disease or by a pre-analytical sample or patient factors [1,2,3].

Urine samples should be stored at room temperature rather than frozen before the analysis. Urine sample analysis should not exceed 60 min post-collection to avoid temperature- and time-dependent modifications, that can influence the accuracy of the results (e.g., time dependent crystal formation) [4,5]. Urinalysis involves the visual assessment of urine color and clarity, determination of urine specific gravity (USG), chemical analysis and urine sediment microscopic evaluation. Urinalysis results should be interpreted alongside the patient’s clinical findings [6,7].

Urine test strips containing colorimetric reagents (dipstick colorimetric tests (DSCT)) are commercially available for the chemistry analysis of urine samples [8,9]. Urine strips can be assessed using semi-automated analyzers or by visual assessment. The chemical reaction in a urine dipstick is based on the color change of a reagent when a specific substance is detected. Color intensity is analogous to the substance concentration being measured. The interpretation of results may vary since color change evaluation depends on the operator’s expertise and subjective grading [10,11]. Semi-automated analyzers are used for reagent test strip interpretation to overcome assessment variations. They are based on the reflectance principle; the more the light is reflected, the lower the concentration of the substance present [12]. Test strips are primarily designed for human use, although they can be used in veterinary medicine, with the exception of the test pads for assessing urine specific gravity (USG), urobilinogen, nitrite and leukocyte esterase, which are unreliable unreliable for animals. The usage of multitest strips is simple, and they generally provide reliable results when the manufacturer’s instructions are followed [13,14]. Among their advantages, dipsticks (dry reagent test strips) are a quick and affordable test to detect semi-quantitative proteinuria along with other physicochemical and cytological parameters [15,16]. Advanced informatics have simplified the analysis workload, offering new technological perspectives for urinalysis [12,17]. Urine dipstick automated analysis can improve accuracy and precision, contributing to diagnosis and better patient management and care [11,18,19]. The purpose of the current study was to compare the visual and automated evaluations of dipstick variables in canine urine samples.

## 2. Materials and Methods

This prospective study included urine samples from client-owned dogs admitted to the Companion Animal Clinic, School of Veterinary Medicine, Aristotle University, over a period of 12 months (January 2021–January 2022) with various presenting complaints. Sampling involved cases presented for diagnostic investigation (complete blood counts, serum biochemistry, urinalysis, diagnostic imaging) or a general health screening (Table 1). Dogs with clinically detectable icterus were excluded from the study (indicating a serum total bilirubin (TBIL) concentration >1.46 mg/dL or 25 μmol/L) [20,21]. Urine samples were obtained either by cystocentesis or “free catch” and evaluated by two experienced individuals (ZP, ES).

Abnormally colored urine samples, caused by hematuria, bilirubinuria or hemoglobinuria, were excluded from further analysis (macroscopic hematuria, >150 RBC/×40) [22], since pigmented urine could interfere with the color reaction reading of the reagent pad [20,23].

The urine sample volume collected was 5–10 mL for “free catch” samples and 2–5 mL for samples collected via cystocentesis. Sterile urine containers were used for collecting “free catch” samples and 5 mL, 21G syringes for cystocentesis (performed under ultrasonographic guidance).

Urinalysis was performed within half an hour of urine sampling. Complete urinalysis involved visual sample inspection (color, clarity and odor), determination of urine specific gravity (USG), dipstick analysis and microscopic evaluation of the urine sediment. Urine specific gravity was determined with a refractometer (Clinical Refractometer Atago T2-Ne, Atago Co., Ltd., Tokyo, Japan). The refractometer was calibrated to 1.000 using distilled water before use [24].

The dipstick analysis was performed on a veterinary urine analyzer URIT-50Vet (URIT Medical Electronic Co. Ltd., Shenzhen, China) using UC VET13 Plus strips and Multistix 10 SG reagent strips (Siemens Healthcare GmbH, Erlangen, Germany) for visual evaluation. Regarding the automated dipstick analysis, calibration and quality control were performed regularly, following the manufacturer’s instructions. The Multistix 10 SG reagent strips (Siemens Healthcare GmbH, Erlangen, Germany) contained test pads for protein, blood, leukocytes, nitrate, glucose, ketone (acetoacetic acid), pH, specific gravity, bilirubin and urobilinogen. The URIT-50Vet analyzer included the following parameters: leukocytes, ketones, nitrite, urobilinogen, bilirubin, protein, glucose, specific gravity, blood, pH value, ascorbic acid (Vitamin C), creatinine, calcium and microalbumin. The dipstick protein, bilirubin, glucose, blood, leukocyte and ketone parameters were evaluated and compared for the two methods (automated and visual inspection) (Table 2 and Table 3). The drip method was used as an application dipstick method in urine samples [25].

Statistical analysis was performed using MedCalc Statistical Software v.14.8.1 (MedCalc Software bvba, Ostend, Belgium). All parameters were evaluated according to their concentration in urine samples, except for bilirubin, which was evaluated with semi-quantitative values. Cohen’s kappa (k) and Passing–Bablok regression were used for the evaluation of the results. In particular, the Passing–Bablok regression was performed to assess the agreement for pH and USG. The inter-rater reliability of the two observers and between visual and automatic analyses for all other variables was measured and weighted by calculation of Cohen’s kappa (κ) coefficient, that varied from 0 to 1. The correlations were ranked as ≤0, poor; 0.1–0.20 = slight agreement; 0.21–0.40, fair agreement; 0.41–0.60, moderate agreement; 0.61–0.80, substantial agreement and 0.81–1, perfect agreement [26].

## 3. Results

**Figure 1:**
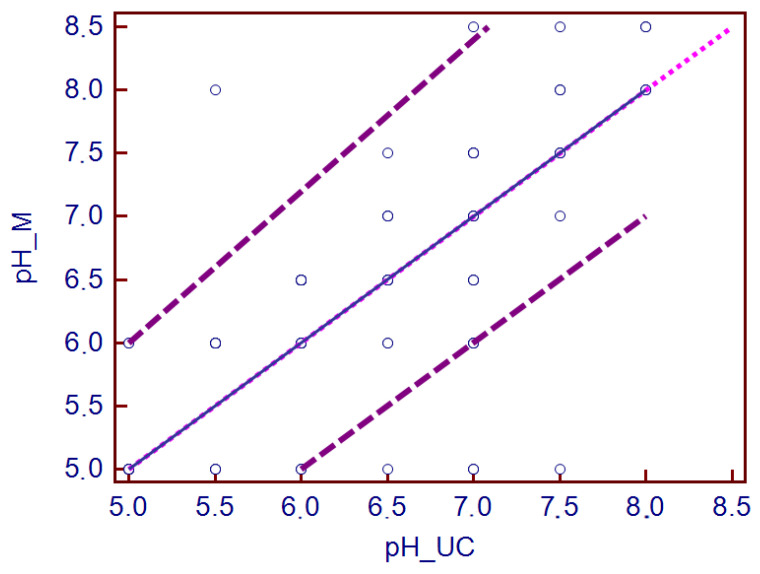
Passing–Bablok regression regarding the comparison of the pH values between the two methods. The plot shows the pH values with the regression line (blue solid line), the confidence interval for the regression line (purple dashed lines) and identity line (x = y, small pink dotted line). PH_UC, automated urine analyzer; pH_M, visual dipstick analysis.

We proceeded with the urine dipstick analysis of 119 canine urine samples; 86 (72%) and 33 (28%) specimens were collected by cystocentesis and free-catch, respectively. Fifty-four (45%) dogs were male and 65 (54%) were female; there were 41 (75%) castrated male dogs and 35 (53%) neutered female dogs. The mean age of the study population on admission was 7.4 years (ranging from 2 months to 17.3 years). According to the visual dipstick reading results, the samples presenting with color reactions were the following: 40 (34%) proteins (15–300 mg/dL), 9 (7.5%) bilirubin (0.4–0.8 mg/dL), 7 (5%) glucose (100–1000 mg/dL), 29 (24%) blood (10–200 cell/μL), 2 (2%) leukocytes (70 cells/μL) and 1 (1%) ketone (15 mg/dL). The automated dipstick analysis revealed 88 (74%) proteins (15–300 mg/dL), 19 (16%) bilirubin (0.5–6 mg/dL), 6 (5%) glucose (50–1000 mg/dL), 25 (21%) blood (10–200 cell/μL), 2 (2%) leukocytes (15–500 cells/μL) and 1 (1%) ketone (15 mg/dL). A binary classification system was used (positive and negative, traces were considered as positive), and the inter-observer agreement was almost perfect (κ = 0.852, 95% confidence interval (CI) 0.656 to 1.000). Subsequently, the inter-rater agreement was κ = 0.627, 95% CI 0.386 to 0.868 when a four-level semi-quantitative scale for rating positives was employed. The median pH value measured by visual dipstick was 6.5 (5.0–8.5, 95% CI) and by automated analyzer was 6 (5.0–8.0, 95% CI). A linear relationship between the pH measurements (*p* = 0.2) of the two methods was noted. Therefore, the Passing–Bablok procedure was considered valid and no significant or proportional systematic errors were observed (Figure 1). Correlation was poor for USG (*p* = 0.01, CI 0.6667 to 1.0000) between the refractometer, the visual dipstick and automated analyzer. A moderate agreement was detected for proteins (κ = 0.431, 95% CI 0.332 to 0.529), bilirubin (κ = 0.434, 95% CI 0.153 to 0.715) and glucose (κ = 0.450, 95% CI 0.160 to 0.739). The agreement presented was substantial for blood (κ = 0.620, 95% CI 0.495 to 0.746) but poor for leukocytes (κ = 0.100, 95% CI −0.100 to 0.300). The agreement was also poor for ketones (κ = −0.006, 95% CI −0.0138 to 0.00258).

## 4. Discussion

The current study compared the urinalysis results obtained with the visual and automated readings of two urine dipsticks. A previous study performed by Bauer et al. (2008) evaluated the results of canine urine samples using a Clinitek 50 strip reader (Siemens Healthcare Diagnostics, Inc., Tarrytown, NY, USA) with Multistix 10 SG dipsticks and Microalbustix reagent strips (Bayer, Newbury UK; now Siemens Medical Solutions Diagnostics GmbH (Dx)) and visual analysis with Combur9 dipsticks (Roche, Basel, Switzerland). In this study, the automated analyses were duplicated and the visual tests were evaluated by two examiners, and these demonstrated an excellent to good concordance comparing the results from the first and second analysis, respectively, with Cohen’s j-values ranging from 0.776 to 1.000. Both dipsticks (visual and automated analyses) showed a good agreement for glucose (j = 0.753), blood (j = 0.793) and protein (j = 0.788), and moderate for bilirubin (j = 0.431) and ketones (j = 0.540) [27]. A previous study revealed good to excellent agreement in all parameters, except for leukocytes (rs = 0.49), in which they validated 101 canine urine results from Aution sticks 10PA and Aution sticks 10EA (both ARKRAY, Kyoto, Japan) and compared them to the semi-automatic urine analyzer Aution Eleven AE-4020 (ARKRAY, Kyoto, Japan) [13]. The results of the current study revealed good agreement only for blood and moderate agreement for the other parameters except for leukocytes and ketones. A possible reason for these results might be the different dipsticks that we used for the visual and automated readings. In general, the leukocyte and specific gravity test pad were not valid in dogs, due to the low sensitivity [8,13,27,28,29]. In contrast to veterinary studies, the sensitivity and specificity were high enough to be a reliable measurement for the detection of pyuria in humans [30,31,32].

In another study, 271 canine urine samples were analyzed using automated and visual dipstick readings with Multistix 10 SG reagent strips (Bayer Diagnostics, Whippany, NJ, USA) and a Bayer Clinitek 50 urine chemistry analyzer (Bayer Diagnostics, Whippany, NJ, USA). The actual glucose concentration was estimated. The correlations between the visual and automated readings and between the automated readings and actual glucose concentration was good; the correlation between the visual analyses and actual glucose concentrations was fair [33]. In our study, in a few samples, glucosuria was detected and moderate agreement was found between the automated and visual readings.

Regarding proteinuria, moderate agreement was revealed between the two methods (automated and visual dipstick evaluations). Two relevant human studies have confirmed the reliability of the results regarding proteinuria between the two methods (automated and visual dipstick tests) [9,25]. More specifically, in the first study, proteinuria was assessed with the strip reader Urisys 1100 analyzer and Chemstrip 10A test strips (both Roche Diagnostics, Laval, QC, Canada) (automated method) and Multistix 10 SG reagent strips (Siemens Healthcare Diagnostics, Inc., Tarrytown, NY, USA) (visual method). Both automated dipstick and visual testing provided reliable results regarding proteinuria in urine samples. The second study assessed the visual evaluation of Multistix 10 SG reagent strips (Siemens Healthcare Diagnostics, Inc., Tarrytown, NY, USA) between two automated methods regarding proteinuria. The two automated readers were a Bayer Clinitek 50 urine chemistry (Siemens Healthcare Diagnostics, Inc., Tarrytown, NY, USA) using Multistix 10 SG reagent strips and a Urisys 1100 analyzer with Chemstrip 10A test strips (both Roche Diagnostics, Laval, QC, Canada). The results showed that proteinuria specificity (visual reading of Multistix 10 SG reagent strips) (Siemens Healthcare Diagnostics, Inc., Tarrytown, NY, USA) was higher (98.4%, *p* < 0.001) than that with the Clinitek 50 strip reader (automated analysis) (Siemens Healthcare Diagnostics, Inc., Tarrytown, NY, USA)/Multistix 10 SG reagent strips (Siemens Healthcare Diagnostics, Inc., Tarrytown, NY, USA) (92.6%, *p* < 0.001) or with the Urisys 1100 analyzer/Chemstrip 10A test strips (both Roche Diagnostics, Laval, QC, Canada)(95.7%, *p* = 0.04) [18]. However, the automated method was more sensitive in proteinuria detection compared to the visual dipstick evaluation. The findings of the current study indicated that semi-quantitative methods may be utilized with caution in the interpretation of proteinuria since the proteinuria severity and semi-quantitative method results showed moderate agreement.

Variations in results are expected in urine colorimetric reaction methods since they can be influenced by individuals’ visual perception and interpretation [34], especially observers with color vision deficiency [35,36,37]. When multiple operators are involved, the use of automated urine dipsticks is ideal, based on a study in a small animal teaching hospital [11]. According to a human study, there were differences between visual (Multistix 10 SG reagent strips, Bayer PLC, Newbury, UK) and dipstick analyzer (Bayer Clinitek 50 urine chemistry analyzer, Bayer PLC, Newbury, UK) readings depending onoperators expertise and subjective grading. These differences in the interpretation of the results may impact clinicians’ decisions regarding therapeutic or management protocols [38]. Consequently, the use of an automated dipstick analyzer could eliminate variations originating from visual dipstick evaluation.

Limitations of the current study included the small number of canine urine samples presenting with glucosuria, ketonuria and pyuria. The different brands of the dipsticks may have an impact on the results. The small number of cases presenting with ketonuria may have affected the inter-rater agreement (poor) between the two observers.

## 5. Conclusions

Apart from the pH analysis, the visual and automated dipstick urinalyses should not be used interchangeably. Multiple urine samples obtained from the same dog during the day should be evaluated using the same method to overcome erroneous results. Automated dipstick urinalysis results were more reliable than visual dipstick inspection, which can be influenced by individuals’ subjective interpretation.

## Figures and Tables

**Table 1:** Conditions diagnosed in the canine study population (n = 119).

Neoplasia	27 (22%)
Infectious diseases	14 (12%)
Neurological disorders	13 (11%)
Cardiopulmonary disorders	12 (10%)
Hematologic and Immunologic diseases	12 (10%)
Hepatobiliary diseases	10 (8%)
Renal diseases	8 (7%)
Gastrointestinal diseases	6 (5%)
General health screening	6 (5%)
Dermatological diseases	5 (4%)
Endocrine diseases	4 (3%)
Pancreatic diseases	2 (1%)

**Table 2:** Semi-quantitative values of Multistix 10 SG parameters.

URIT-50Vet							
Analyte	Semi-Quantitative Symbol and Concentration						
Proteins	Semi-Quantitative	-	trace	+1	+2	+3	
	mg/dL	0	15	30	100	300	
Bilirubin	Semi-Quantitative	-		+1	+2	+3	
	mg/dL	0		0.5	2	6	
Glucose	Semi-Quantitative	-	trace	+1	+2	+3	+4
	mg/dL	0	5	100	250	500	≥1000
Blood	Semi-Quantitative	-	trace	+1	+2	+3	
	cells/μL	0	10	25	80	200	
Leukocytes	Semi-Quantitative	-	trace	+1	+2	+3	
	cells/μL	0	15	70	125	500	
Ketone	Semi-Quantitative	-	trace	+1	+2	+3	
	mg/dL	0	5	15	40	≥80	

**Table 3:** Semi-quantitative values of URIT-50Vet analyzer parameters.

Multistix 10 SG Reagent Strips							
Analyte	Semi-Quantitative Symbol and Concentration						
Proteins	Semi-Quantitative	-	Trace	+1	+2	+3	+4
	mg/dL	0	15	30	100	300	≥2000
Bilirubin	Semi-Quantitative	-		+1	+2		+3
Glucose	Semi-Quantitative	-	Trace	+1	+2	+3	+4
	mg/dL	0	100	250	500	≥1000	≥2000
Blood	Semi-Quantitative	-	Trace	+1	+2	+3	
	cells/μL	0	10	25	80	200	
Leukocytes	Semi-Quantitative	-	Trace	+1	+2	+3	
	cells/μL	0	15	70	125	500	
Ketone	Semi-Quantitative	-	Trace	+1	+2	+3	+4
	mg/dL	0	5	15	40	≥80	≥160

## Data Availability

Not applicable.
